# Development of a simple method for measuring tedizolid concentration in human serum using HPLC with a fluorescent detector

**DOI:** 10.1097/MD.0000000000028127

**Published:** 2021-12-10

**Authors:** Yasuhiro Tsuji, Miki Numajiri, Chika Ogami, Fumihiro Kurosaki, Aoi Miyamoto, Takahiko Aoyama, Hitoshi Kawasuji, Kentaro Nagaoka, Yoshiaki Matsumoto, Hideto To, Yoshihiro Yamamoto

**Affiliations:** a Center for Pharmacist Education, School of Pharmacy, Nihon University, Funabashi, Chiba, Japan; b Department of Medical Pharmaceutics, Graduate School of Pharmaceutical Sciences, University of Toyama, Toyama, Japan; c Laboratory of Chemical Biology, Graduate School of Pharmaceutical Sciences, University of Toyama, Toyama, Japan; d Laboratory of Clinical Pharmacokinetics, School of Pharmacy, Nihon University, Funabashi, Chiba, Japan; e Department of Clinical Infectious Diseases, Graduate School of Medicine and Pharmaceutical Sciences, University of Toyama, Toyama, Japan.

**Keywords:** high-performance liquid chromatography, pharmacokinetics, target concentration intervention, tedizolid

## Abstract

The objective of the present study was to develop a method to measure tedizolid (TZD) concentration for studying target concentration intervention, pharmacokinetics, and pharmacodynamics of TZD. We established a high-performance liquid chromatography-fluorescence detector assay to measure the TZD concentration in serum for clinical application. Chromatographic separation was carried out on a 5 μm octadecyl silane hypersil column 150 mm × 4.6 mm. The mobile phase consisted of 0.1 M phosphoric acid and methanol (60:40, pH 7.0). Detection was performed at 300 nm and 340 nm for the excitation and emission wavelengths, respectively. The average retention times of TZD and the internal standard were 12.9 and 8.8 min, respectively. High linearity was exhibited over a concentration range of 0.025 to 10.0 μg/mL for TZD (*R*^2^ > 0.999). The intra- and inter-assay accuracies of TZD were 99.2% to 107.0% and 99.2% to 107.7%, respectively. The lower limit of quantitation and the lower limit of detection for TZD measurement were 0.025 and 0.01 μg/mL, respectively. The extraction recoveries of TZD were 100.4% to 114.1%.

The high-performance liquid chromatography method developed in this study could separate the analytes with a single eluent (isocratic system), within a total run time of 15 min. Both TZD and IS were well separated, without interference from the peaks. Sharp peaks were observed in the chromatograms; problems such as double peaks, shoulder peaks, and broadened peaks were not observed. The proposed method showed acceptable analytical performance and could be used to evaluate serum TZD concentrations in patients.

## 1 Introduction

To ensure optimal antimicrobial chemotherapy in patients infected with methicillin-resistant *Staphylococcus aureus* (MRSA), target concentration intervention has been recommended for anti-MRSA agents.^[1]^ The measurement of anti-MRSA agent concentration in human serum is a routine practice for monitoring patients treated with vancomycin, teicoplanin, and arbekacin in Japan. However, this is generally not performed with other anti-MRSA agents.

Linezolid (LZD)^[2]^ and tedizolid (TZD)^[3]^ are synthetic antibacterial agents belonging to the oxazolidinone family that have a unique mechanism of action. In recent years, pharmacokinetic (PK) and pharmacodynamic (PD) studies of LZD have been actively conducted.^[4]^ The clinical efficacy and side effects of LZD depend on serum LZD concentration.^[5]^ Target concentration intervention is an important approach for optimizing LZD activity in patients infected with MRSA.^[6]^ TZD is the most recent anti-MRSA agent, and it was approved in Japan in 2018; thus, the PKPD of TZD is still less frequently reported. However, as TZD also belongs to the oxazolidinone class, it is likely to develop the same drug concentration-dependent clinical effects and side effects as that of LZD.^[7]^

As a pioneering effort, the development of a method to measure the serum TZD concentration could make a significant contribution in the effective treatment of MRSA-infected patients. The purpose of the present study was to establish a high-performance liquid chromatography-fluorescence detector (HPLC-FL) assay system that can easily measure the serum TZD concentration for clinical application.

## 2 Materials and methods

### 2.1 Ethics

This study was conducted with the approval of the ethics committee of University of Toyama (approval number: R2012133 revised) and Nihon University (School of Pharmacy, approval number: 20–005 and 20–012).

### 2.2 Evaluation of theoretical concentration range

To determine the concentration range of the calibration curve, a pharmacokinetic simulation was performed based on the pharmacokinetic parameters reported in a previous study.^[8]^ A one-compartment distribution with zero-order infusion (intravenous) or first-order absorption (peroral) and elimination model was applied to predict TZD concentrations for 7 days. For the simulation, 200 mg of TZD was administered every 24 h by intravenous or oral route. The rate of infusion was assumed to be 1 h. PK parameter values needed for the prediction of TZD concentration were randomly generated using average and standard deviation values in a previous study^[8]^ following a log-normal distribution, and 1000 simulations were performed using these values. Simulation and graphical analyses were performed using R version 3.6.3.

### 2.3 Chemicals

**Figure 1:**
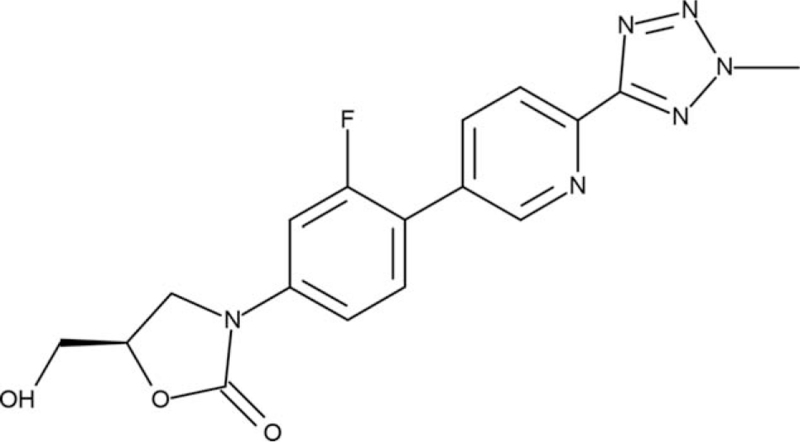
Chemical structure of tedizolid (370.17 g/mol).

The chemical structure of TZD is shown in Figure 1. TZD bulk powder (purity: ≥98%) was purchased from LKT Laboratories, Inc (Saint Paul, MN). L-tryptophan methyl ester hydrochloride was purchased from Tokyo Chemical Industry Co, Ltd (Tokyo, Japan) and used as an internal standard (IS). Pooled drug-free serum from a healthy volunteer was purchased from Kohjin Bio Co, Ltd (Saitama, Japan) as the blank. Dipotassium hydrogen phosphate, potassium dihydrogen phosphate, and dimethyl sulfoxide were purchased from FUJIFILM Wako Pure Chemical Corporation (Osaka, Japan). Acetonitrile and methanol were of HPLC-grade and purchased from Kanto Chemical Co, Inc (Tokyo, Japan). Ultra-pure water was obtained from an ultra-pure water production device (Arium mini; Sartorius Göttingen, Germany).

### 2.4 Chromatography conditions and instruments

Chromatographic separation was performed using an isocratic elution. The HPLC system consisted of a Prominence HPLC unit (20A series, Shimadzu Co, Kyoto, Japan) comprising a binary pump, column oven, autosampler, and FL detector. Data were evaluated using the LabSolutions software. Chromatographic separation was carried out on an octadecyl silane hypersil column (Cadenza 5CD-C18, 150 mm × 4.6 mm, 5 μm; Imtakt Co, Kyoto, Japan), protected by a guard column (Cadenza 5CD-C18 Guard Column 5 × 2 mm, Imtakt Co, Kyoto, Japan). The mobile phase was 0.1 M phosphoric acid (adjusted to pH 7 with 61 mM dipotassium hydrogen phosphate and 39 mM potassium dihydrogen phosphate) and methanol (0.1 M phosphoric acid/methanol: 60/40 (vol)). TZD was measured by the fluorescence intensity with the excitation and emission wavelengths for the optimum detection set to 300 and 340 nm, respectively. The temperatures of the column oven and auto-sampler were set to 40°C and 4°C, respectively. The injection volume was 20 μL. The flow rate was constant at 1.0 mL/min.

### 2.5 Calibration and sample preparation

#### 2.5.1 Calibration and quality control samples

A 250 μg/mL TZD aqueous solution was prepared by dissolving 1 mg of pure TZD powder in dimethyl sulfoxide to a final volume of 4 mL. The solution was serially diluted with ultrapure water. Twenty microliters of these dilutions were added to 80 μL of drug-free human serum to obtain final TZD concentrations of 0.01, 0.025, 0.05, 0.1, 0.5, 1.0, 5.0, and 10.0 μg/mL. Simultaneously, the quality control (QC) samples of TZD also were prepared to obtain a final TZD concentration of 0.025 (assumed lower limit of quantitation [LLOQ]), 0.1 (low QC), 5.0 (medium QC) and 10.0 (high QC) μg/mL.

#### 2.5.2 Sample preparation

Twenty-five microliters of 50 μg/mL IS (L-tryptophan methyl ester hydrochloride aqueous solution) was added to 100 μL of each TZD serum sample. One hundred microliters of acetonitrile was added to each sample and vortexed for 30 s to deproteinize the serum. Samples were left at normal temperature (15–30°C) for 10 min and centrifuged for 5 min at 14,000×*g*. Then, 150 μL of the filtered supernatant was collected in a vial and transferred to an HPLC autosampler.

### 2.6 Analytical method validation

Validation of the developed analytical method for selectivity, calibration curve linearity, accuracy, and precision was performed in accordance with the U.S. Food and Drug Administration (FDA) guidelines.^[9]^

#### 2.6.1 Selectivity

To ensure the absence of interfering peaks from endogenous matrix components, selectivity was investigated by examining the separation of TZD and IS from the serum matrix components of blank human serum.

#### 2.6.2 Calibration curve

The calibration curves were constructed by plotting the TZD peak height, divided by the IS peak height. Linear regression analysis method was used to calculate the linearity of the calibration curves. The LLOQ of TZD was defined as the lowest concentration of calibration standards that could be measured with acceptable accuracy and precision. The signal-to-noise (S/N) ratios (>10) were also defined to determine the LLOQ of the HPLC method. The lower limit of detection (LLOD), defined as the S/N ratio, was >3.

#### 2.6.3 Accuracy and precision

The intra- and inter-day accuracy and precision were evaluated using serum TZD samples. Accuracy was expressed as a percent bias of each QC concentration, and precision was expressed as the relative standard deviation of each QC concentration (Equation 1).


$$Accuracy(\%)=\frac{Measured\;concentration}{Theoretical\;concentration}\times 100$$

$$\mathrm{Pr}ecision(relative\;s\tan dard\;deviation\%)=\frac{S\tan dard\;deviation}{Average}\times 100$$

Acceptance limits were defined as accuracy between 85% and 115% and precision of <15%, except at the LLOQs, which were defined as accuracy between 80% and 120% and precision of <20%.

#### 2.6.4 Recovery

The extraction recoveries of TZD and IS were calculated by comparing the peak heights of the high-, medium-, and low-extracted QC samples with those of the pre-spiked standards. The recovery was determined by analyzing the QC samples at three concentration levels and the IS for intra- and inter-day.

## 3 Results

### 3.1 Application of theoretical concentration range

The lower 2.5% of the predicted TZD concentration at day 1 (after 24 h) was 0.09 μg/mL for intravenous (iv) and 0.11 μg/mL for peroral (po), and the upper 97.5% was 3.79 μg/mL for iv and 2.09 μg/mL for po. The lower 2.5% of the predicted TZD concentration at day 7 (after 168 h) was 0.10 μg/mL for iv and 0.16 μg/mL for po, and the upper 97.5% was 4.32 μg/mL for iv and 3.10 μg/mL for po.

### 3.2 Chromatography and calibration

**Figure 2:**
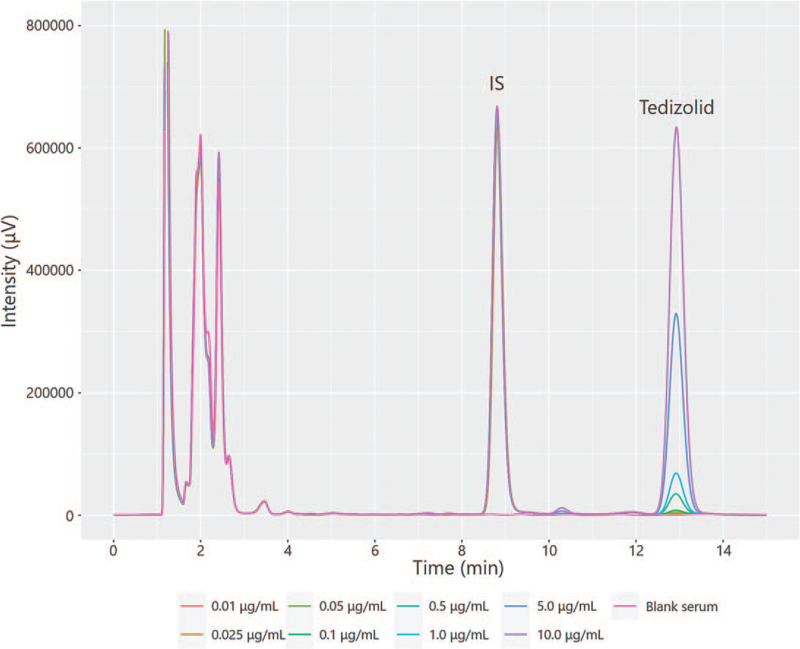
High-performance liquid chromatography chromatograms of tedizolid (TZD) in serum samples. The peaks of TZD were detected at an average 12.9 (standard deviation (SD) 0.007) min and those of L-tryptophan methyl ester hydrochloride as internal standard (IS) were detected at an average 8.8 (SD 0.005) min.

HPLC chromatograms of serum samples spiked with TZD (0.01–10.0 μg/mL) and IS are shown in Figure 2. The average retention times of TZD and IS were 12.9 and 8.8 min, respectively. The equations for the calibration curve for TZD are shown in Table 1. The calibration curves were constructed based on seven samples with different concentrations of 0.025 to 10 μg/mL for each assay by plotting the TZD peak height a divided by the IS peak height. High linearity was exhibited over a concentration range of 0.025 to 10.0 μg/mL for TZD (*R*^2^ > 0.999). The S/N of LLOQ (0.025 μg/mL) in all assays was >10.0 and that of LLOD (0.01 μg/mL) in all assays was >3, but a few exceeded 10.

**Table 1:** Intra- and inter-assay calibration curve parameters for determination of tedizolid.

Intra-assay (n = 3)			Inter-assay (n = 3)		
Slope	Intercept	Correlation coefficient	Slope	Intercept	Correlation coefficient
0.0702 ± 0.0562	0.0032 ± 0.0015	0.9996	0.0976 ± 0.0002	0.0033 ± 0.0013	0.9996

### 3.3 Analytical method validation

The results of the accuracy and precision tests of the developed method for analyzing TZDs are shown in Table 2. The intra- and inter-assay accuracies of TZD were 99.2% to 107.0% and 99.2% to 107.7%, respectively. The intra-and inter-assay precisions, except for the LLOQ, were 0.5% to 3.2% and 0.3% to 4.1%, respectively; intra- and inter-assay precisions for the LLOQ were 17.0% and 15.3%, respectively.

**Table 2:** Intra- and Inter-assay accuracy and precision for the determination of tedizolid.

	Intra-assay (n = 3)			Inter-assay (n = 3)		
Theoretical concentration (μg/mL)	Observed value^a^ (μg/mL)	Accuracy (%)	Precision (%)	Observed value^a^ (μg/mL)	Accuracy (%)	Precision (%)
0.025 (LLOQ)	0.026 ± 0.004	105.7	17.0	0.027 ± 0.004	107.7	15.3
0.100 (low QC)	0.107 ± 0.003	107.0	3.2	0.105 ± 0.004	105.3	4.1
5.00 (medium QC)	5.15 ± 0.09	102.9	1.7	5.15 ± 0.06	103.1	1.2
10.00 (high QC)	9.92 ± 0.05	99.2	0.5	9.92 ± 0.03	99.2	0.3

The results of the recovery tests for the three QC levels are presented in Table 3. The intra- and inter-assay extraction recoveries of TZD were 101.9% to 111.9% and 100.4% to 114.1%, respectively. The intra- and inter-assay extraction recoveries of IS were 103.4% to 105.7% and 101.9% to 108.4%, respectively.

**Table 3:** Recovery of tedizolid and internal standard in human serum.

Concentration added (μg/mL)	Intra-assay (n = 3)		Inter-assay (n = 3)	
	Tedizolid	IS	Tedizolid	IS
0.100 (low QC)	111.9 ± 3.3 (2.9)	104.9 ± 5.7 (5.5)	114.1 ± 0.9 (0.8)	108.4 ± 1.6 (1.5)
5.00 (medium QC)	102.7 ± 1.2 (1.2)	105.7 ± 0.1 (0.1)	100.4 ± 2.6 (2.6)	101.9 ± 3.5 (3.4)
10.00 (high QC)	101.9 ± 2.9 (2.8)	103.4 ± 3.3 (3.2)	103.7 ± 2.1 (2.0)	104.0 ± 4.6 (4.4)

## 4 Discussion

TZD phosphate is a prodrug of TZD. On administration of TZD phosphate to humans, the prodrug undergoes rapid hydrolysis to TZD. The administered TZD phosphate in plasma following single or multiple administrations was not detected for 72 h, and all were below the LLOQ.^[10]^ TZD is a pharmacologically active anti-microbial agent, but TZD phosphate is pharmacologically inactive. Therefore, the target of quantification in this study was TZD and not TZD phosphate.

We investigated the theoretical concentration range of TZD using PK simulation. The LLOQ was set to a value <1/2 of the lower 2.5% of the predicted TZD concentration. The maximum concentration of the calibration curve was set to a value greater than twice the upper 97.5% of the predicted TZD concentration. The range of the calibration curve established in this study was considered to be capable of measuring the TZD concentrations in TZD-treated patients.

L-tryptophan methyl ester hydrochloride used as the IS is esterified with methyl of L-tryptophan, which inherently exists in the human body. There was a possibility that IS was present in the drug-free human serum. We compared the chromatograms of the serum samples without IS to serum samples with IS. No peaks of IS were found in the serum samples without IS (data not shown). This suggests that L-tryptophan methyl ester hydrochloride is an effective IS for measuring TZD concentrations using HPLC-FL.

The HPLC method developed in this study could separate the analytes with a single eluent (isocratic system), within a total run time of 15 min. Both TZD and IS were well separated without interference from the peaks. Sharp peaks were observed in the chromatograms; problems, such as double peaks, shoulder peaks, and broadened peaks, were not observed. The extent of recovery of the analyte and IS should be consistent and reproducible. The current data indicated that the sample preparation method was satisfactory, and both the compounds, TZD and IS, were recovered at high yields. The results of validation for selectivity, calibration curve linearity, accuracy, precision, and recovery of the analytical method developed in the present study were all within the range of reference values in compliance with the FDA guidelines.

A comparison of our method for the measurement of TZD with those reported in previous studies is presented in Table 4. Previously, methods of detecting TZD using HPLC-tandem mass spectrometry have been reported.^[8,11–16]^ To improve the current analytical capability by HPLC with a limited budget, we investigated the conditions for an efficient and accurate determination of TZD using the HPLC-FL method. In addition, several groups have reported methods for detecting TZDs using the HPLC-ultraviolet detector (UV) method.^[17–19]^ In this study, the HPLC-FL method was applied to develop a reasonably rapid method in a practical range. LLOQ and LLOD in this study with HPLC-FL method were defined as 0.025 μg/mL and 0.01 μg/mL, respectively. The LLOQ values obtained with our method for TZD in the present study were similar to the values obtained in previous studies with HPLC-UV; however, the measurement accuracy in the low concentration range could be improved. To the best of our knowledge, this is the first study to determine TZD concentration using HPLC-FL.

**Table 4:** Comparison of methods for measuring tedizolid in this study with those reported in previous studies.

Authors	Year	Method of measurement	LLOQ (LLOD)	IS	Samples
Present study:		HPLC (fluorescence detector)	0.025 μg/mL (0.01 μg/mL)	L-tryptophan methyl ester hydrochloride	Serum
Previous studies:					
Housman ST et al^[11]^	2012	LC-MS/MS	0.005 μg/mL	Stable isotope labeled tedizolid	Serum, BAL fluid
Sahre M et al^[17]^	2012	HPLC (Ultraviolet detector)	0.05 μg/mL	Not shown	Plasma
Ong V et al^[12]^	2014	LC-MS/MS	(0.005 μg/mL)	Stable isotope labeled tedizolid	Plasma
Flanagan S et al^[10]^	2014	LC-MS/MS	0.005 μg/mL	Not shown	Plasma, urine
Bradley JS et al^[13]^	2016	LC-MS/MS	(0.005 μg/mL)	Not shown	Plasma
Yu HC et al^[14]^	2016	UPLC-MS/MS	0.005 μg/mL	Diazepam	Plasma
Deshpande D et al^[15]^	2017	LC-MS/MS	0.1 μg/mL	Linezolid D-3	Serum, plasma
Park AYJ et al^[16]^	2018	LC-MS/MS	0.001 μg/mL	Not shown	Plasma, sputum
Stainton SM et al^[18]^	2018	HPLC (No description of detector)	0.2 μg/mL	Not shown	Plasma
Dorn C et al^[19]^	2020	HPLC (Ultraviolet detector)	0.03 μg/mL	Not shown	Plasma

This study had several limitations. First, we were unable to measure the concentration of TZD using actual cases in which it was administered. Therefore, the fraction of unbound plasma protein was not evaluated based on the relationship between the total and unbound concentrations. It is generally accepted that only the concentrations of unbound plasma proteins are responsible for pharmacologically beneficial activities and side effects. It is necessary to establish testing methods for unbound TZD concentrations. Another limitation is that the details of the system suitability are not shown. However, all the measuring equipment used in this research undergo regular maintenance.

In conclusion, we developed a simple method for measuring TZD concentration in humans using HPLC-FL, which showed acceptable analytical performance.

## Acknowledgments

The study was supported by Japan Society for the Promotion of Science (JSPS) KAKENHI Grant Numbers JP19K08950 and JP20K07189, Nihon University Multidisciplinary Research Grant for 2020.

## Author contributions

**Conceptualization:** Yasuhiro Tsuji, Chika Ogami, Yoshihiro Yamamoto.

**Data curation:** Miki Numajiri, Chika Ogami.

**Formal analysis:** Miki Numajiri, Chika Ogami.

**Funding acquisition:** Yasuhiro Tsuji, Yoshihiro Yamamoto.

**Investigation:** Miki Numajiri, Fumihiro Kurosaki, Aoi Miyamoto, Takahiko Aoyama.

**Methodology:** Chika Ogami, Aoi Miyamoto, Takahiko Aoyama, Hideto To.

**Project administration:** Yasuhiro Tsuji, Yoshiaki Matsumoto, Yoshihiro Yamamoto.

**Resources:** Yasuhiro Tsuji, Yoshiaki Matsumoto, Hideto To, Yoshihiro Yamamoto.

**Software:** Chika Ogami.

**Supervision:** Yasuhiro Tsuji, Yoshiaki Matsumoto, Yoshihiro Yamamoto.

**Validation:** Hitoshi Kawasuji, Kentaro Nagaoka.

**Visualization:** Yasuhiro Tsuji, Chika Ogami.

**Writing – original draft:** Yasuhiro Tsuji.

**Writing – review & editing:** Yasuhiro Tsuji, Miki Numajiri, Chika Ogami, Fumihiro Kurosaki, Aoi Miyamoto, Takahiko Aoyama, Hitoshi Kawasuji, Kentaro Nagaoka, Yoshiaki Matsumoto, Hideto To, Yoshihiro Yamamoto.
